# The Schoolmaster Very Much Abroad

**Published:** 1892-11-12

**Authors:** 


### PASSING TOPICS.

### THE SCHOOLMASTER VERY MUCH ABROAD.

Notwithstanding our reverence for all constituted authority, we altogether sympathise with the distressed father who, when recently summoned by the School Board inspector, told the magistrate that he preferred to have his children educated at home, until they were old enough to go to school, and we must express considerable surprise that so reasonable a desire should have provoked the presiding magistrate of the South-Western Police-court to the extraordinary pronouncement that "private people could not educate their children, because education is a science."

The defendant is not singular in his plea. The objection to schools has been shared by a great many people whose intelligence and ability to form a trustworthy opinion are unquestionable, and so far from discouraging parents from attempting at least the earlier education of their children, this course ought to be urged upon them as a duty. It is to its neglect that we owe many of the grievous and all too common lapses of conduct which distinguish the youthful "students" and "scholars" of the present day. Summed up in a word, we have an era of teaching without training, and in place of the fostering care which was at one time the birthright of every child of decent parentage, and the home influences which count for much in a comprehensive and rational plan of education, we have a woefully exaggerated estimate of the power and importance of mere "book-learning," coupled with a partial or an entire neglect of the culture, moral and social, which alone makes character—

> "The truths on which depends our main concern,
> That 'tis our shame and misery not to learn."

And if Cowper could be severe upon those high-class schools, which he dealt with so mercilessly in his "Tirocinium," what, we may well ask, would be the opinion of his or any refined mind concerning such herding places as the unfortunate defendant in the case we have mentioned is now compelled by magisterial decision to have recourse to for his children? Schools in which the teachers' purview of duty is limited to the time passed in the school-room, where the best traditions of school life are altogether wanting, and a code of school honour has no soil in which to grow.

If anyone desires to learn in what estimation Board schools are held, let him collect reliable data of the efforts made by the inhabitants of any given neighbourhood to secure themselves against the erection of a school-house in their midst. We doubt if the objections to a fever hospital are more uncompromising or more persistently urged. And this opposition is due less to the fact that the playground is uncomfortably distinguished by a display of shrieking and senseless inanities instead of the pursuit of healthful recreation, than because the eruption of the children into the roadways when school is over means a recurring exhibition of tumult, insolence, and such indecency of act and language as description is powerless to exaggerate. The poor man in question declared that his objection to send his children to the Board school was chiefly due to the "abominable language used by some of the scholars," and whatever the law may say, right and reason are upon the side of the objector, whose protest is entitled to profound respect. The subject is no doubt a difficult one, and schools must be. But educational machinery, if properly designed, ought to be capable of turning out something better than youths a tenth-part educated and wholly savages.

Agricola.

